# Admission to day stay early parenting program is associated with improvements in mental health and infant behaviour: A prospective cohort study

**DOI:** 10.1186/1752-4458-6-11

**Published:** 2012-08-13

**Authors:** Heather Rowe, Sonia McCallum, Minh Thi Hong Le, Renzo Vittorino

**Affiliations:** 1 The Jean Hailes Research Unit, School of Public Health and Preventive Medicine, Monash University, Monash, VIC 3168, Australia; 2 Tweddle Child and Family Health Service, 53 Adelaide Street, Footscray, VIC 3011, Australia

**Keywords:** Parenting, Infants, Mental health, Health services

## Abstract

**Background:**

Australia’s Early Parenting Services support families and intervene early in mental health problems in parents. The Victorian Early Parenting Strategy, a platform for government policy recommended a stronger evidence base for early parenting services. Tweddle Child and Family Health Service (TCFHS) is a not-for-profit public sector early parenting centre, which provides residential, day stay, home visiting and outreach programs. This study aimed i) to examine the health, social circumstances and presenting needs of clients attending the Tweddle Day Stay Program (DSP) with infants under 12 months old and ii) to assess the parent mental health and infant behaviour outcomes and the factors associated with program success.

**Methods:**

A cohort of clients was recruited prior to admission and followed-up 8 weeks after discharge. Data were collected using standardised measures in a study specific questionnaire at baseline, participant’s Tweddle records and a follow-up telephone interview. Health, social circumstances and presenting needs of clients were described. Changes in parents’ symptoms of depression and infants’ sleep and settling between admission and follow-up were calculated. Multiple regression analyses were conducted to examine factors associated with changes in primary outcomes.

**Results:**

Of the total 162 clients who were eligible and invited to participate, 115 (72%) were recruited. Parents admitted to the DSP had worse general self-reported physical and mental health than community samples. Infants of DSP participants were no more likely to be premature or have low birth weight, but significantly more unsettled than other community samples. Participants’ mental health and their infants’ behaviours were significantly improved after DSP admission. In multivariate analysis, higher depression score at baseline and greater educational attainment were significantly associated with improvements in parents’ mental health. Worse unsettled infant behaviours and longer time between discharge and follow up were significantly associated with improvements in infant sleep and settling.

**Conclusions:**

Tweddle DSPs appear to respond effectively to the needs of families presenting with substantial physical and emotional health morbidity and a range of vulnerabilities by treating parental mental health and infant behaviour problems together. DSPs offer important potential benefits for prevention of more serious family problems and consequent health care cost savings.

## Background

### Health policy context

The mental health of parents of infants in Australia is a growing clinical and public health priority. Australia’s National Perinatal Depression Initiative [1] has provided funding to each state and territory to improve care for women who are at risk of or experiencing depression in pregnancy and in the first year after birth. The initiative aims to enhance recognition of mental health problems in primary care services, foster better networks of support groups for new mothers and enhance care and support in community-based, as well as specialist psychological and acute inpatient services. In addition to implementing the national initiative, the Victorian State Government has identified early childhood is a priority. Recent policy and legislative changes are intended to promote earlier intervention and more timely and effective services for vulnerable children and families [2].

Victoria’s early parenting sector provides day stay, residential, group and home-based programs focussing on infant health and development, promotion of family wellbeing and parent-infant emotional attachment. This sector is well placed to respond to both the national perinatal mental health and Victorian early childhood agendas. The Victorian Early Parenting Strategy (VEPS) [2] provides the policy platform for Victorian early parenting services. The strategy is consistent with the public health model of family care, which is underpinned by universal services such as the Maternal and Child Health nursing service, available free to all families with infants under the age of five [2]. The second tier of the model includes the early parenting services, which are designed as intensive secondary services and which parents attend on a voluntary basis when universal services are insufficient for their needs. These services are focussed on prevention and early intervention. They are intended to reduce the need for referral to tertiary services, which constitute the third tier of the model of service provision [2].

The VEPS is based on growing evidence of the importance of investing in the early years of life and on recognition of the central role of the early parenting sector in achieving this goal. The strategy identifies three key focus areas. First, to strengthen the integration of the early parenting services within the Victorian Child and Family service system; second, to enhance the range of service responses to changing community needs and third, to build service capacity to promote quality and innovation [2]. A review of existing early parenting services in Victoria was conducted as part of the VEPS. The Strategy recommended that, in order to make clear decisions about future service directions, a stronger evidence base about the outcomes of early parenting services be developed [2].

Residential programs offered by early parenting services are internationally-unique, structured admissions to assist parents of infants with unsettled behaviour and feeding problems [3]. There is no doubt that these residential programs in Victoria and other Australian states constitute an important component of comprehensive mental health care for young families [4]. Up to 25% of mothers admitted meet diagnostic criteria for major depression, 25-32% meet criteria for a current anxiety disorder [5,6] and severe fatigue is universal [7]. Admission is consistently associated with significant and sustained improvements in maternal mental health and infant behaviour [4,8].

A similar structured program of one day duration, designed to assist parents of infants with sleep and feeding difficulties, is offered in the Day Stay Program. Compared with residential programs, less is known about the characteristics and presenting needs of families attending day stay programs or the outcomes of these services. The effectiveness of a Melbourne metropolitan Day Stay Program was tested in a randomised controlled trial. Mental health status, parental confidence and infant behaviours all improved significantly after admission [9]. Another recent prospective investigation of women admitted to a day stay early parenting program in Western Australia showed that one month after attendance, compared to a comparison sample, women who attended the day stay program had significantly better confidence and competence, but not mental health as assessed by the Edinburgh Postnatal Depression Score (EPDS) [10][11]. These inconsistent findings should be interpreted with caution and might be explained by methodological factors. For example, in the first study, allocation to groups was not concealed and attrition in the control group was higher than in the intervention group [9]. In Hauck and colleagues’ [11] study ,the comparison group was a convenience sample of volunteers and the outcome analyses were not adjusted for worse baseline mental health in the admitted group.

In order to contribute to the VEPS recommendation to enhance the evidence base and as part of its commitment to a regular review and priority-setting agenda, the Board of Tweddle Child and Family Health Service commissioned an independent investigation of its Day Stay Program in 2010.

### Aims of the study

This study was conducted in order to assist the Tweddle Board with their organisational strategic planning and to contribute to the evidence base about current Victorian Early Parenting Centre (EPC) service provision. The aims of the study were: i) to examine the health, social circumstances and presenting needs of clients attending the Tweddle DSP and ii) to assess the parent mental health and infant behaviour outcomes of the program and the factors associated with program success.

## Methods

Ethics approval was obtained from the University of Melbourne Human Research Ethics Committee (Date of approval: 13 July, 2010; Ethics ID: 1033203).

### Study setting

#### Tweddle child and family health service

Tweddle Child & Family Health Service (TCFHS) is a not-for-profit public sector early parenting centre, established in the 1920s and now funded through the Victorian Government Department of Human Services. Tweddle’s residential, day stay, home visiting, outreach programs aim to facilitate learning and parenting skill development in parents of babies and children up to 4 years old. TCFHS is part of the second tier of services, and programs are offered by experienced and highly skilled maternal and child health nurses, midwives, and early childhood professionals free-of-charge to parents from a wide range of socioeconomic and cultural backgrounds, living in metropolitan and rural areas. TCFHS prioritises provision of services to vulnerable or isolated families and to parents of infants and young children assessed as at risk.

#### Tweddle day stay program

The Day Stay Program (DSP) service was established in 1992. DSPs are managed by Tweddle or jointly with other organisations. These programs have good local recognition and are highly regarded and used [2]. A goal of the program is to link families to supports within their community including mental health, maternal and child health, general practice and family support agencies.

The objectives of the DSP are that parents explore alternative ways of managing their parenting concerns, receive coaching in interpreting their child’s cues; explore factors that have contributed to their loss of confidence and be referred to services in their community [12].

Parents self-refer to the DSP by telephoning the service directly. Experienced nurses conduct an assessment interview and triage to the appropriate Tweddle service, based on severity of presenting needs, age of child and personal and socioeconomic circumstances.

#### Theoretical model of DSP

Groups of 2 to 4 families are admitted together to the 7-hour program. Parents are assisted individually to establish short-and long-term goals for their child and themselves. A health promotion model of practice is used in which one-to-one and group learning opportunities and supported practice are provided. Educational strategies include discussion, self-directed learning, counselling, coaching, demonstration and didactic presentations. Participants learn about infant behaviour management strategies to promote settling and sleep, optimal infant nutrition and feeding practices, infant development and behaviour, managing parental fatigue and promoting emotional well being, parent - infant relationships, safety and play [13].

### Study design

The study was a prospective longitudinal cohort, or single group pre-and post-test design. Participants were assessed twice: once prior to admission to the DSP and once 4 – 8 weeks after discharge from the program.

### Participants

Clients were identified by the TCFHS intake team as being eligible for the study if they were 18 years old or over, with English proficiency sufficient to give informed consent to participate and complete a written questionnaire and a telephone interview, and had accepted a place in the DSP with an infant under 12 months old.

### Sample size

Based on the normal distribution, a sample size of 97 at outcome, provides 95% confidence that the true population prevalence of study parameters will lie between ± 5% of the prevalence estimates observed in the study [14].

### Data sources

#### Participant data

1. Tweddle Client Record

As part of admission procedures, all clients provide personal and health information, which is held electronically in the computerised triage program Client Assessment and Intake System (CAIS) and in paper records of registration and maternal and infant histories.

2. Study-specific questionnaire

This collected demographic information including postcode and participant and baby’s date of birth, general health, mental health and infant behaviour.

3. Follow-up computer assisted telephone interview (CATI)

The interview assessed general and mental health, infant behavior, health service use, assessment of child’s behavior and parenting enjoyment since attending the DSP.

#### Standardised measures

Standardised, validated, published, self-report measures were used to enable comparison of the study sample with relevant population norms.

1. General health

General self-reported health (GSRH) was assessed using the single question “In general, would you say your health is: excellent/ very good/ good/ fair/ poor?” derived from the SF-36 [15]. This question is a good predictor of mortality and healthcare utilization, comparable to other multi-item measures [16].

2. Mental health

i) The EPDS is a widely-used 10-item self-rating scale for screening for probable depression, using 4 response options (0–3) and yielding a total score of 0–30 [10]. It has been validated in Australia against diagnostic interviews. A score of greater than 12 yields a sensitivity of 100%, a specificity of 95.7% and a positive predictive value of 69.2% for depression [17].

ii) The Kessler 6 is a six-item self-rating scale [18] used to detect serious mental illness. Five response options for each question (scored 1 – 5) generate a total score between 6 and 30. A higher score indicates more distress and a score of 19 and over signifies a high risk of mental disorder [19].

3. Infant behaviour

The Baby Behaviour Scale (BBS) [20] is a parent self-report measure of average duration of infant crying, frequency of night time waking, ease of soothing and settling and number and length of day time sleeps in a 24-hour period during the last two weeks. Maternal confidence is assessed with a single question. These eight items yield good internal consistency (Cronbach Alpha = 0.7 [20]). Scores on 8 individual items are summed, total scores range between 0 and 21, higher scores indicate more unsettled infant behaviour.

#### Comparison population data

Four sources of comparison data were used.

1. 2009 Victorian Child Health and Wellbeing Survey

A Victorian statewide survey was conducted by the Data Outcomes and Evaluation Division, Department of Education and Early Childhood Development in 2009. This study had a response fraction of 75% and used a CATI to survey primary caregivers of 5025 randomly selected Victorian children aged under 13 years [21].

2. Birth in Victoria 2007 and 2008

The most recent summary data available for all births in Victoria [22].

3. Tweddle Residential Service in 2004

Presenting needs and outcomes of 79 women admitted to the Tweddle residential service [4,23].

4. Two community samples

a) A survey assessing the mental health of 12,361 women recruited from 43 health services across Australia at 6–8 months postpartum [24].

b) Mental health and infant behaviour reported by 800 women attending local government immunisation clinics in the Tweddle catchment area with their four month old infants [20].

### Procedure

#### Recruitment

All eligible women who were offered and accepted a place at a Tweddle DSP were posted a letter of invitation to participate, a plain language statement, a consent form, a contact detail form and a brief study specific questionnaire with their standard pre-admission pack.

#### Participation and follow-up

Those agreeing to participate signed a standard consent form, provided contact details and completed the study specific questionnaire. Clients placed their sealed envelope (whether materials had been completed or not) in a box provided during attendance at the DSP. Trained telephone interviewers contacted participants within 4–8 weeks of attendance of the DSP to assess their continued willingness to participate and arrange a suitable time to conduct the interview. Where necessary, at least three repeated attempts were made to reach participants by telephone.

#### Participant compensation

To compensate for participants’ time and inconvenience, a shopping voucher to the value of AUD 25 was posted to those who completed all stages of the study.

#### Data extraction from client record

Data were extracted from participants’ client record and entered into a study-specific standardised data extraction tool by a member of Tweddle staff and the research team working together.

### Data management and analysis

The sources of data, measures used and response options for sociodemographic characteristics, heath and circumstances, reproductive history, maternal mental health, partner relationship, social support, infant characteristics, health service use, parenting and infant behaviours are presented in Additional file 1.

#### Data analyses

Data were analysed in SPSS v 19 [25]. Total scores and proportions above relevant cut-off scores on standardised scales were computed for the EPDS [10] and Kessler 6 [18] measures.. Clinically significant symptoms, defined as EPDS scores over 12 (for clinical samples) and over 9 (for community samples) [10] and Kessler 6 scores over 19 [26] were computed and entered as categorical variables. Items on the BBS [20] were recorded on individual 3 or 4-point scales. Individual item scores were summed and a total score computed as a continuous variable. Individual items were reported as frequencies (n; %) and total score as a continuous variable.

Normality tests were conducted on continuous data. Internal consistencies of numerical measures were established and reported as Cronbach’s alpha statistic. Descriptive statistics were computed using mean (SD) for continuous variables and frequency distributions for categorical data. Non-parametric one-sample binomial and chi-square tests were used to establish all significant differences in baseline variables from population-based comparison data. Baseline characteristics of participants retained in the study were compared with those lost to follow-up using Pearson chi-square and Fisher’s exact test for multinomial and binomial categorical variables respectively; Mann–Whitney tests for non-normally distributed and t-tests for normally distributed continuous variables.

The primary outcomes were defined as changes in mean EPDS and BBS scores between baseline and follow-up (Time 2 – Time 1) and reported as mean differences (95% confidence interval for the difference). Employment status was re-coded into a binary variable as employed versus not in paid employment; mode of birth as caesarean or assisted versus spontaneous vaginal; birth weight as low (<2500grams) versus normal (>2500grams) in regression analyses. Multiple regression analyses were conducted in order to examine the factors associated with the primary outcomes. First, factors potentially associated with outcomes as hypothesised were checked in bi-variable analyses. Variables with p-values < 0.2 [27]were included in the models. Model statistics including regression coefficients, 95% CIs, and p-values are presented. Negative coefficients indicate greater improvements in the outcomes and positive coefficients indicate deteriorations in outcomes.

## Results

The study took place between 1 August 2010 and 30 October 2011. Recruitment and retention of participants are described in 1. Of 162 clients who were eligible, invited to participate and attended the DSP, 116 (72%) participated. Owing to the complexities of triage and admission in this busy clinical setting, not all women who attended the DSP were offered the opportunity to participate in the study. However, there was no systematic bias in the process of recruitment. Of those who completed the baseline assessment, 103 (89%) completed the follow-up interview. There were no significant differences in baseline characteristics between participants who were successfully followed up and those who were either lost to contact or withdrew from the study (data not shown).

**Figure 1:**
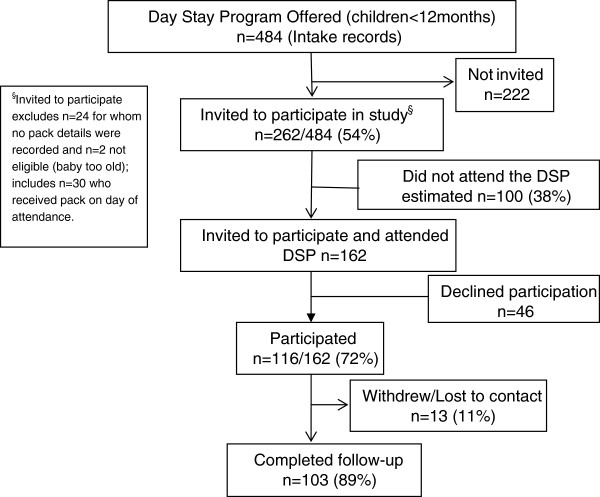
Recruitment and participation.

### Health, social circumstances and presenting needs of clients attending the Tweddle DSP

#### Sociodemographic characteristics

In general, participants were as likely to be born in Australia as Victorian parents, but less likely than those admitted to the residential program in 2004. Parents attending Tweddle DSPs were more likely to be married or in a de facto relationship and have higher educational attainment than a Victorian community sample of mothers of infants and a sample from the Tweddle residential program.. Study participants were also less likely to be in paid employment than the Tweddle residential service sample and to depend on a health care or pension card than parents in Victoria. Participants were drawn from all socioeconomic sectors in the community (Table 1).

**Table 1:** Sociodemographic characteristics

**Characteristic**	**Number of valid responses**	**Sample**	**Comparison**	**p**
Mean (SD) age (years)	115	32.3 (4.9)	30.8^2^ 32.2 (4.9)^3^	0.02. 0.91
Aboriginal/ Torres Strait Islander n (%)	86	0	1.0^2^	0.35
Born in Australia n (%)	114	86 (75.4)	72.8^2^	0.30
			86.6^4a^	<0.001
			85^3^	0.01
Language spoken at home n (%)	114			
Other than English		14 (12.3)	12^3^	0.5
Marital status n (%)	109			
Married		77 (70.6)	73.4^2^	<0.001
De facto		28 (25.7)	13.5^2^	
Separated		2 (1.8)	0.4^2^	
Single		2 (1.8)	12.0^2^	
Education attainment n (%)	113			
Primary				
Secondary		29 (25.7)		
Tertiary		84 (74.3)	17.6^4a^	<0.001
(Post-secondary)			67.0^3*^	0.06
Current employment status n(%)	113			
No		63 (55.8)	31	<0.001
No (study part time)		1 (0.9)	4.0^3^	
Yes (maternity leave)		34 (30.1)	27.0^3^	
Yes		15 (13.3)		
Full-time		3 (20.0)		
Part-time		12 (80.0)		
Pension/ Health care card				
Participant (n (%))	108	17 (15.7)	27.0^1^	0.01
Partner (n (%))	103	3 (2.9)		
Socioeconomic Position^d^ (IRSAD) (n (%))	114			
Lowest 20%		11 (9.6)	7.6^4b^	0.08
21-40%		6 (5.3)	10.4^4b^	
41-60%		16 (14.0)	8.5^4b^	
61-80%		50 (43.9)	48.8^4b^	
Highest 20%		31 (27.2)	24.7^4b^	

^1^ 2009 Victorian Child Health and Wellbeing Survey.

^2^ Births in Victoria 2007 and 2008 [22].

^3^ Tweddle Residential Service 2004 [4,23].

^4a^ Community Sample: Buist 2008 [24].

^4b^ Community Sample: McCallum 2011 [20].

#### General and reproductive history

Parents attending the DSP rated their health as significantly worse than other Victorian parents but somewhat better than a sample of women admitted to the Tweddle residential program. Almost three quarters reported poor/ very poor/ extremely poor sleep, about the same proportion were first time parents, and almost a third (n = 34; 29%) had health problems, most arising from birth and breastfeeding. More than half reported experiencing at least one distressing life event in the previous 12 months, including unemployment, separation from partner, eating disorder, miscarriage, financial difficulties, alcohol or substance abuse, domestic violence and the death of someone close. Almost three quarters of participants were fully or partly breastfeeding, which, at a mean infant age at admission of approximately 6 months, suggests that breastfeeding in this sample is relatively common compared to the general population (Table 2).

**Table 2:** Health and reproductive history

**n (%)**	**Number of valid responses**	**Sample**	**Comparison**	**p**
**Maternal Health**				
General health question n(%)	116		47.0^3^	0.13
Excellent/Very good		61 (52.6)		
Good		50 (43.1)		
Fair/ Poor		5 (4.3)		
How would you describe your sleep pattern? n(%)	116			
Unsure		6 (5.2)		
Good		2 (1.7)		
Average		21 (18.1)		
Poor		59 (50.9)		
Very poor		15 (12.9)		
Extremely poor		13 (11.2)		
Experienced distressing life events in the last 12 months	113	64 (56.6)	46.0^3^	0.02
n (%)				
**Reproductive history**				
Number of pregnancies n(%)	113			
One		66 (58.4)	33.4^2^	<0.001
Two		30 (26.5)	29.7^2^	
Three or more		17 (15.2))	36.9^2^	
Total children n(%)	114			
One		82 (71.9)		
Two		27 (23.7)		
Three or more		5 (4.4)		
Adverse pregnancy events n(%)	114	24 (21.1)		
Mode of birth n(%)	115			0.04
Normal		73 (63.5)	55.4^2^	
Caesarean		35 (30.4)	30.6^2^	
Assisted		7 (6.1)	14.0^2^	
Continuing concerns n(%)	112	16 (14.3)		
Postnatal complications n(%)	111	34 (29.3)		
Breastfeeding n(%)	113			
Fully		27 (23.9)		
Partly		46 (40.7)	61^3^*	

^1^ 2009 Victorian Child Health and Wellbeing Survey.

^2^ Births in Victoria 2007 and 2008 [22].

^3^ Tweddle Residential Service 2004 [4,23].

^4a^ Community Sample: Buist 2008 [24].

^4b^ Community Sample: McCallum 2011 [20].

* “At least some breastfeeding”.

#### Mental health and social support

The mental health of the sample was assessed using self-report measures. The study sample mean EPDS score and the proportions scoring more than 9 and more than 12 (clinically significant symptoms of depression in community and clinical samples respectively) were both significantly higher, indicating worse mental health, than two community comparison samples [20,24]. However the degree of psychological distress in this sample was not as severe as in the sample admitted to Tweddle residential programs [4].

The K6, designed to detect high risk of serious mental disorder, detected a similar proportion of participants at risk of serious mental illness as in Victorian parents who participated in the Victorian Child Health and Wellbeing Survey [21] (Table 3). Overall, the results show that clients of Tweddle DSPs are experiencing psychological distress symptoms of a magnitude that warrants clinical attention. 

**Table 3:** Participant mental health

**Variables**	**Number of valid responses**	**Sample**	**Comparison**	**p**
EPDS * score	114			<0.001
Mean (SD)		8.9 (4.5)	5.5^4b^;	<0.001
			11.3^3^	<0.001
n (%) >9		53 (46.5)	16.7^4b^;	<0.001
			15.4^4a^;	<0.001
n (%) >12		24 (21.1)	7.6^4a^; 39.0^3^	<0.001 <0.001
Mean Kessler 6 *score				
Total (mean(SD))	115	11.6 (3.5)		
n (%) at risk of mental disorder (>18)		4 (3.5)	3.7^1^	0.5
Current feelings of depression or anxietyn (%)	116	28 (24.1		
Previously feelings of depression or anxiety n (%)	116	32 (27.6)		

^1^ 2009 Victorian Child Health and Wellbeing Survey.

^2^ Births in Victoria 2007 and 2008 [22].

^3^ Tweddle Residential Service 2004 [4,23].

^4a^ Community Sample: Buist 2008 [24].

^4b^ Community Sample: McCallum 2011 [20].

The availability of social support is known to act protectively or to increase risk of mental health problems in the life stage when caring for an infant [28]. More than half (n = 64, 55%) of the study participants rated the support that they were receiving from their partner in the work of infant care and household management as low or very low. Few participants (n = 14, 12.1%) were receiving substantial support from friends or family and even fewer still (n = 10, 8.6%) endorsed their community as a source of support. These findings are consistent with those in the sample admitted to the residential service [4]. It appears that attendance at the DSP is a means of addressing substantial need for additional parenting support and countering feelings of social isolation (Table 3).

#### Infant characteristics at admission

Consistent with Tweddle’s triage policy, which prioritises DSP places for younger infants, those admitted to DSPs were on average younger than those admitted to the residential unit. Admitted infants were no more likely to be premature or have low birth weight than other Victorian born infants. Two thirds of infants were reported as having experienced a medical condition since the birth and a substantial number of parents reported an infant developmental concern (unspecified) in their infants. The distribution of infant age of introduction of solid foods indicates that the most parents (n = 55, 85%) are doing so prior to the recommended 6 months of age [29]. Although this is likely to be consistent with community norms, the wide disparity may indicate parental confusion because of recent changes in the recommended age for introduction of solid foods into an infant’s diet (Table 4). 

**Table 4:** Infant characteristics

**Variables**	**Number of valid responses**	**Sample**	**Comparison**	**p**
Sex	114			
Male n(%)		67 (58.8)	51.5^2^	0.07
Female n(%)		47 (41.2)	48.5^2^	
Mean (SD) infant age (weeks)	115	26.5 (11.74)	31 (11.7)^3^	<0.001
Gestational age n(%)	113			
Pre-term (<37 weeks)		5 (4.4)	7.9^2^6.0^3^	0.12 0.31
Birth weight n(%)	114			
<2500 g		6 (5.3)	6.5^2^	0.37
>2499 g		108 (94.7)	93.5^2^	
Illnesses or medical conditions since birth n(%)	115	76 (66.1)		
Health development concerns n(%)	108	18 (16.7)		

^1^ 2009 Victorian Child Health and Wellbeing Survey.

^2^ Births in Victoria 2007 and 2008 [22].

^3^ Tweddle Residential Service 2004 [4,23].

^4a^ Community Sample: Buist 2008 [24].

^4b^ Community Sample: McCallum 2011 [20].

### Prior health service

Participants reported contact with a variety of health services prior to attending Tweddle DSP. Of the 7 listed services, participants endorsed a mean (SD) = 2.2 (0.98) (range 0 – 5) services which they had attended since the birth of the baby. Contact with a MCH nurse was near universal (n = 101, 90%) and a smaller proportion (n = 91, 81%) had consulted a general practitioner.

### Presenting concerns

#### Parenting

No comparison data are available for these questions, but almost all participants described themselves as happy with the job of being a parent (n = 110, 94.8%) and their relationship with their child (n = 106, 91.4%). However, a smaller proportion (n = 88,76%) reported being happy with their child’s behaviour and less than half (n = 49, 48%) were enjoying parenting.

#### Infant sleep and settling

Infants admitted to DSPs (n = 106) were reported as exhibiting significantly more unsettled behaviour (mean; SD = 10.9; 2.96) than those in the community (mean = 6.1; mean difference = 4.8 (95%CI: 4.26; 5.40); p < .001) [20]. Infants were reported as having shorter daytime sleeps, crying inconsolably for longer periods, more difficult to soothe, waking more often at night and more difficult to settle back to sleep, having fewer daytime sleeps, and more difficult to look after than infants in the community (p < 0.001 for all items). Only 81% of the parents (n = 94) attending the DSP reported feeling confident about looking after their babies compared to 97% of the community sample [20]. The BBS at admission yielded a Cronbach alpha of 0.62.

### Parent mental health and infant behaviour outcomes of the program and factors associated with program success

The median time to follow-up was 7 (range 2 – 25) weeks. There was a need, for staffing reasons, to interview a small group (n = 5) before 4 weeks after discharge and another group (n = 27) were unable to be interviewed until after 8 weeks.

#### Mental health

All indicators of mental health showed significant improvements between baseline and follow-up (Table 5). Mean (95% CI) EPDS scores at follow-up (5.0 (4.3; 5.7)) had significantly decreased to community norms (5.5; p > .05 [20]) as had the proportion of participants with EPDS scores in the clinical range (>12) (5.8% cf 7.6% [24]). The factors associated with improvements in mental health after admission in bi-variable analyses (data not shown) were modelled in multiple regression. Of all the factors included in the model, two made a significant contribution to improvement in mood. First, participants whose EPDS score at baseline was higher, had a significantly greater improvement in depression symptoms at follow-up. Second, compared with participants with lower educational attainment (Year 12 and less) those with tertiary qualifications had significantly greater improvements in mood after admission (Table 6). 

**Table 5:** **Mental health at admission and at follow up (n = 103**^**+**^**)**

**Measure**	**Admission (T1)**		**Follow-up (T2)**		**Difference (T2-T1)**	
	N*	**Mean (95%CI)**	**N**	**Mean (95%CI)**	**N**	**Mean (95%CI)**
EPDS score**	101	8.7(8.1; 9.7)	103	5.0(4.3; 5.7)	101	−3.7(−4.5; -2.9)
Kessler 6 score**	102	11.4(10.9;12.2)	103	9.8(9.3; 10.4)	102	−1.5(−2.1; -.8)

* N: Valid responses.

^+^participants who completed baseline and follow-up assessments; *some missing data.

**Kessler 6: Cronbach α = 0.733; EPDS: α = 0.799.

**Table 6:** Factors associated with difference (T2-T1) in EPDS scores between baseline (T1) and follow-up (T2)

**Factor**	**Coefficient**	**95 % CI**	**p-value**
Education (1:Tertiary; 0:Year 12 and less)	−1.87	−3.64 ; -0.1	0.03
EPDS score at baseline	−0.59	−0.77 ; -0.41	<0.001
Work(1: Employed; 0: not in paid employment)	−0.81	−2.25 ; 0.64	0.3
English at home (1: Yes; 0: No)	−1.54	−3.95 ; 0.88	0.2
Distressing event (1: Yes; 0: No)	−1.74	−3.91 ; 0.44	0.1
Baby Behaviour score at baseline	−0.08	−0.35 ; 0.2	0.5
Birthweight (1: Low; 0: Normal)	1.01	−1.48 ; 3.51	0.4
Time to follow-up (month)	−0.67	−1.76 ; 0.41	0.2
Mother's age	0.47	−2.96 ; 3.91	0.8
Baby's age	−0.19	−0.46 ; 0.08	0.2

#### Infant behaviour

Infant behaviour had also improved significantly. The mean (95% CI) reduction in BBS score was 2.4 (1.8; 3.0), but the mean (95% CI) score at follow-up (8.4 (7.7; 8.9)) remained significantly higher than in 4 month old infants in the community (6.1; p < 0.001) [20]. The BBS at follow up yielded a Cronbach alpha of 0.67. Responses to individual BBS items at baseline and follow-up were compared. There had been significant improvements on all items, except the number of daytime sleeps. Most infants were still having two or three sleeps during the day, which is appropriate in this age group. Importantly, parental confidence had increased significantly (data not shown).

The factors associated with improvements in infant behaviour in bi-variable analyses were modelled in multiple regression. Two factors made a significant contribution to the change score. Infants who had more unsettled behaviour (high BBS score) at baseline showed greater improvements in sleep and settling at follow-up than those with less problematic behaviour at admission. There also appears to have been improvement in infant behaviour over time: the longer the elapsed time between DSP attendance and the follow-up interview, the greater the improvement in infant behaviour that had occurred (Table 7).

**Table 7:** Factors associated with difference (T2-T1) in Baby Behaviour scores between admission (T1) and follow-up (T2)

**Factor**	**Coefficient**	**95 % CI**	**p-value**
Baby Behaviour score at baseline	−0.39	−0.61 ; -0.18	<0.001
Time to follow-up (month)	−1.04	−1.87 ; -0.21	0.02
Breastfeeding (1: Fully; 0: Partly/ none)	−1.17	−2.73 ; 0.39	0.14
Working (1: Employed; 0: not in paid employment)	0.84	−0.28 ; 1.96	0.14
EPDS score at baseline	−0.06	−0.2 ; 0.07	0.36
Birth (1: c/s/assisted; 0: spontaneous vaginal)	−0.74	−1.95 ; 0.48	0.23
Birthweight (1: Low; 0: Normal)	0.94	−1.28 ; 3.16	0.40
Baby medical condition (1: Illness; 0: None)	−0.74	−1.89 ; 0.41	0.21
Baby age (month)	−0.04	−0.3 ; 0.22	0.77

### Health service use

A stated aim of the DSP at Tweddle is to link families into other health and social services in their local communities. Almost one quarter (n = 22, 21%) of participants had been referred by staff during their DSP admission to another service, including MCH nurse, GP, paediatrician, social worker, counsellor or psychologist and the Tweddle residential program. Some participants had been given *beyondblue* pamphlets for their partners to use. However, only half of these participants had taken up their referral. Reported reasons for not attending their MCH nurse included dissatisfaction with their nurse, too long a gap between visits, the inability to get a date for an appointment and improvements their infant‘s night-time sleep and settling. A partner did not take up a referral to a psychologist because of his reluctance to talk about his feelings with someone unknown to him. Reasons for not attending the Tweddle residential program as suggested related to the perception that this service is for more problematic cases.

## Discussion

This study examined the mental health of parents attending a short-admission early parenting service in Victoria Australia and gives important insights into the nature and severity of their presenting problems and the value of the service. However, the study was limited by the need to collect some data from client record, rather than with study specific questions. This was necessary to maximise client participation and minimise the burden on participants and staff. The “single group pre- and post-test” design limits the conclusions that can be drawn about the effect of the DSP program on the outcomes of interest. This is because improvements in indicators of parent and infant wellbeing might be expected to occur spontaneously with the passage of time, growing infant developmental capacities and recovery from the birth. However, the longitudinal design, high participation and retention, the collection of data using relevant standardised measures with which comparisons with population-based data could be made, from a sample which is large enough to provide statistically precise estimates of sample characteristics. However, the findings should be generalised to all parents who use the DSP with caution, because not all women who attended the DSP were offered the opportunity to participate in the study.

The results provide the service with important opportunities for review. The most striking finding is the level of need that is being addressed by the DSP. Many participants reported current health problems and coincidental adverse life events. On average their physical and mental health status and their infants’ unsettled behaviour were significantly worse than community norms. The significantly elevated symptoms of anxiety and depression prior to admission had, by follow up, declined to community norms and the infants were on average significantly more settled. Greater improvements were experienced by clients who were more symptomatic at admission. Together, the findings confirm the value of integrating assistance for infant sleep and settling into mental health services for parents of infants [3].

The program also appears to be more beneficial for people with more experience in formal learning settings, who gained an almost two fold greater benefit from participation than those with fewer years of formal education. As well as the generally protective effect on mental health of socioeconomic status and better education, it is possible that better educated participants were able to make more effective and sustained use of the psycho-education during admission than those with less education. The service is currently reviewing the model of care, including simplifying written materials and providing more supported practice of new skills and opportunities for individual explanation during the admission. Longer elapsed time to follow up was also associated with greater improvements in infant behaviour. This might be explained by growing infant maturity with the passage of time or resulted from the time taken for parents to establish new patterns of infant care before the benefits became apparent.

The finding that DSP clients were better educated, in higher status occupations and less likely to be born overseas than in the general population could be explained by possible, unfamiliarity with community services of overseas-born Australians, and the preferential triage of the more disadvantaged to the residential program at the service. However, it emphasizes that more advantaged groups can also be vulnerable at this phase of life.

### Implications

Changes to the service model, which include increasing visibility and access for vulnerable and culturally and diverse families and modifying the educational components to meet the needs of clients with less formal education are recommended. The low uptake of referral to other services after discharge is of potential concern given that a key objective of the DSP is to improve links to other community resources. Participants expressed dissatisfaction with other available services but their reasons for lack of engagement with these services warrant exploration.

The potential prevention of more serious of mental health-related problems associated with the DSP early intervention is an important finding. This would be likely to reduce the costs associated with treatment, productivity losses, and to reduce impaired quality of life of families, which sometimes may be immeasurable [30]. The cost effectiveness of the DSP service remains to be investigated.

## Conclusions

The findings of this study suggest, notwithstanding sub-optimal integration with other services, that the Tweddle DSPs form an important part of the spectrum of services for parents of infants in Victoria. DSPs appear to respond effectively to needs that are more complex than can be met in universal MCH services but generally do not require residential admission. The DSPs are responding to high level of physical and emotional health need and a range of vulnerabilities. DSPs offer important potential benefits for prevention of more serious family problems and consequent health care cost savings.

## Competing interests

The authors declare that they have no conflict of interests. The study was funded by the Tweddle Board and staff assisted with the implementation. However, study design, data collection, data analysis, interpretation of results and manuscript preparation were conducted independently by researchers. Comments from Tweddle staff on the results were sought; but the university researchers retained authority over all stages of the research and reporting process.

## Authors' contributions

HR designed the study in collaboration with SMc and RV. SMc and ML collected and analysed the data. SMc and RV extracted medical record data. HR drafted the manuscript with assistance from ML and all authors reviewed and revised the manuscript prior to submission.

## Supplementary Material

Additional file 1 ** Data sources, measures and response options**[10]**,**[18]**,**[20] Click here for file
