# Supplementary material for: Admission to day stay early parenting program is associated with improvements in mental health and infant behaviour: A prospective cohort study
Source: Int J Ment Health Syst. 2012 Aug 13;6:11. doi: 10.1186/1752-4458-6-11 (PMC3464700; doi:10.1186/1752-4458-6-11)
Supplement: Additional file 1 — Data sources, measures and response options[10],[18],[20] [file 1752-4458-6-11-S1.doc]

**Additional file 1 Data sources, measures and response options**

| **Measures** | **Response options** | **Data sources** | | | | | |
| --- | --- | --- | --- | --- | --- | --- | --- |
|  |  | **Baseline** | | | | | **Follow up** |
|  |  | Study specific questionnaire | CAIS | Tweddle Client record | | | Telephone interview |
|  |  |  |  | Mother history | Child history | Registration form |  |
| **Sociodemographic characteristics** | | | | | | | |
| Maternal Age (years) | Free text | **√** |  |  |  |  |  |
| Aboriginal/ Torres Strait Islander origin | Yes/ No |  |  |  |  | **√** |  |
| Country of birth | Free text |  |  |  |  | **√** |  |
| Language spoken at home | English/ Other |  |  |  |  | **√** |  |
| Marital status | Married/ De facto/ Separated/ Single |  |  |  |  | **√** |  |
| Education attainment | Primary/ Secondary/ Tertiary (Post-secondary) |  |  | **√** |  |  |  |
| Current employment | No/ No (study part-time)/ Yes (maternity leave)/  Yes: Full time/ Part time |  |  | **√** |  |  |  |
| Work description  ANZSCO | Free text; classified according to the ANZSCO |  |  | **√** |  |  |  |
| Pension/Health care card | Yes/ No |  |  |  |  | **√** |  |
| Socioeconomic Position^a^ (Postcode (IRSAD)) | Residential Postcode | **√** |  |  |  |  | **√** |
| **Maternal Physical Health** | | | | | | | |
| General self-reported health | Excellent/ Very good/ Good/ Fair/ Poor | **√** |  |  |  |  | **√** |
| Sleep pattern | Unsure/ Good/ Average/ Poor/ Very poor/ Extremely poor |  |  |  |  |  |  |
| Distressing life events in the last 12 months | Yes/ No (none; unemployment; separation; eating disorder; miscarriage; financial difficulties; moving house; physical illness; domestic violence; alcohol/ drug addiction; death of someone close or other) |  | **√** | **√** |  |  |  |
| **Reproductive history** | | | | | | | |
| Number of pregnancies | Free text |  |  | **√** |  |  |  |
| Number of children | Free text |  |  | **√** |  |  |  |
| Adverse pregnancy events | miscarriage, stillbirth, sudden infant death, illness or injury |  |  | **√** |  |  |  |
| Mode of birth | Normal/ Caesarian/ Assisted |  | **√** |  |  |  |  |
| Other prolonged concerns since pregnancy and/ or labour? | Yes/ No |  |  | **√** |  |  |  |
| Postnatal complications | Yes/ No |  |  | **√** |  |  |  |
| Breastfeeding | Fully/ Partially or none |  |  |  | **√** |  |  |
| **Maternal Mental Health** | | | | | | | |
| EPDS | See [10] | **√** |  |  |  |  | **√** |
| Kessler 6 | See [18] | **√** |  |  |  |  | **√** |
| Current self-report depression or anxiety | Yes/No |  | **√** |  |  |  |  |
| **Measures** | **Response Options** | **Data Sources** | | | | | |
|  |  | **Baseline** | | | | | **Follow up** |
|  |  | Study specific questionnaire | CAIS | Tweddle Client Report | | |  |
|  |  |  |  | Mother history | Child history | Registration form | Telephone interview |
| Previous self-report depression or anxiety | Yes/No |  | **√** |  |  |  |  |
| **Partner, family and community support** | | | | | | | |
| Partner support | Very low/ Low/ Average/ High/ Unsure |  | **√** |  |  |  |  |
| Family/friend support | Very low/ Low/ Average/ High/ Unsure |  | **√** |  |  |  |  |
| Community support | Very low/ Low/ Average/ High/ Unsure |  | **√** |  |  |  |  |
| **Infant Factors** | | | | | | | |
| Infant’s date of birth | Free text | **√** |  |  |  |  | **√** |
| Infant’s sex | Male/ Female |  | **√** |  |  |  |  |
| Gestational age at birth | Free text (weeks) |  | **√** |  |  |  |  |
| Birth weight | Free text; classified as ≥2500gr or <2500gr |  | **√** |  |  |  |  |
| Infant health | None/ One or more illnesses (respiratory problems, skin rashes; eczema, thrush, ear infection, gastro, allergies, nappy rash, reflux or other condition since birth) |  |  |  | **√** |  |  |
| Infant development | Yes/ No |  |  |  | **√** |  |  |
| Introduction of solid foods | Age of commencement (months) |  |  |  | **√** |  |  |
| **Health services and medication use** | | | | | | | |
| Health service visited  MCHN  General Practitioner  Paediatrician  Psychologist/ counselor  Social/ Child care worker | Yes/ No |  |  | **√** |  |  | **√** |
| Current medication use | Yes/ No |  |  | **√** |  |  | **√** |
| Referral Source | Self/ MCH Nurse/ Midwife/ Friend, family or other |  | **√** |  |  |  |  |
| Tweddle attendance decision | Self/ Partner/ Jointly/ Other |  |  | **√** |  |  |  |
| **Concerns about parenting and infant behaviour** | | | | | | | |
| Perceived quality of parenting role | Happy/ Neutral/ Unhappy/ Unhappy/ Very unhappy/ Extremely unhappy |  | **√** |  |  |  | **√** |
| Quality of parent-infant relationship | Happy/ Neutral/ Unhappy/ Unhappy/ Very unhappy/ Extremely unhappy |  | **√** |  |  |  | **√** |
| Perception of infant’s behaviour | Happy/ Neutral/ Unhappy/ Unhappy/ Very unhappy/ Extremely unhappy |  | **√** |  |  |  | **√** |
| Parenting enjoyment | Not enjoying it at all/ Not enjoying very much/ Mostly enjoying it/ Very much enjoying it/ Extremely enjoying it |  |  | **√** |  |  | **√** |
| **Infant sleep and settling** | | | | | | | |
| Baby Behaviour Scale | See [20] | **√** |  |  |  |  | **√** |
